# Which combination cause less inflammatory response during laparoscopic hysterectomy? Ligasure plus monopolar cautery or harmonic plus bipolar cautery?

**DOI:** 10.12669/pjms.39.5.7668

**Published:** 2023

**Authors:** Emrah Toz, Ahkam Goksel Kanmaz, Abdurrahman Hamdi Inan, Ibrahim Karaca, Suna Yıldırım Karaca, Ahmet Demir

**Affiliations:** 1 Emrah Toz Associate Professor, Department of Gynecology and Obstetrics, Izmir Tepecik Education and Research Hospital, Izmir, Turkey; 2 Ahkam Goksel Kanmaz Associate Professor, Department of Gynecology and Obstetrics, Izmir Tepecik Education and Research Hospital, Izmir, Turkey; 3 Abdurrahman Hamdi Inan Associate Professor, Department of Gynecology and Obstetrics, Izmir Tepecik Education and Research Hospital, Izmir, Turkey; 4 Ibrahim Karaca Associate Professor, Department of Gynecology and Obstetrics, Izmir Cigli Training and Research Hospital, Izmir, Turkey; 5 Suna Yıldırım Karaca Obstetrics/Gynaecology, Department of Gynecology and Obstetrics, Izmir Tepecik Education and Research Hospital, Izmir, Turkey; 6 Ahmet Demir Associate Professor, Department of Gynecology and Obstetrics, Izmir Tepecik Education and Research Hospital, Izmir, Turkey

**Keywords:** Ligasure, Harmonic, Laparoscopy, Hysterectomy, Neutrophil-lymphocyte ratio, Platelet-lymphocyte ratio, Mean platelet volume-lymphocyte ratio

## Abstract

**Objective:**

We aimed to compare the inflammatory response with alterations in hemogram parameters, in patients who underwent laparoscopic hysterectomy and *bilateral salpingo*-*oophorectomy* for benign gynecologic conditions with ligasure plus monopolar cautery or harmonic plus conventional bipolar cautery.

**Methods:**

Patients who underwent Laparoscopic hysterectomy with bilateral *salpingo*-*oophorectomy* between January 2017 and January 2022 for benign gynecologic pathology were identified. Patients were divided into two group, according to instruments used during surgery. Instruments were used according to surgeons preference. Preoperative and postoperative in the first 24 hours hematocrit (HCT), WBC, trombocyt, neutrophil- lymphocyte (NLR), platelet-lymphocyte (PLR) ratio, mean platelet volume- lymphocyte ratio (MPVLR) and red cell distribution width- platelet ratio (RPR) values were compared.

**Results:**

During study period, a total of 462 patients underwent hysterectomy for benign gynecologic pathology. After exclusion, 212 patients were included in the study. İn the study group, 147 patients were operated with ligasure plus monopolar electrocauter and 65 with harmonic scalpel plus bipolar electrocautery. İn the postoperative period, regardless of the procedure, WBC and RPR count increase, hematocrit and trombocyt decrease in both group but the inflammatory markers lymphocyte count, neutrophyl, NLR, PLR and MPVLR count changed less in the harmonic plus bipolar cautery group which shows less inflamatuar response in this group.

**Conclusions:**

Ligasure plus monopolar cautery group compared with harmonic plus bipolar cautery group cause more inflammatory changes in complete blood count values. However, further studies are needed to show whether these changes in laboratory findings affect clinical situations.

## INTRODUCTION

Hysterectomy is the second most common surgery for women after cesarean section and the abdominal removal of the uterus is the most commonly used method by surgeons.^1^ Hysterectomy can also be done through the vagina or as a laparoscopic or robot-assisted surgery. Regardless of the type of surgery used, hysterectomy is a major surgical procedure and studies have shown that these kind of major surgical trauma promotes an immunologic dysfunction that predisposes the patient to significant morbidity. Leukocyt and neutrophil count increase after the surgery, whereas lymphocytes and thrombocytes decreased because of the effects of various hormones and cytokines.^2^,^3^ Due to the surgical stress, the blood levels of cytokines, such as interleukin-1 (IL-1), tumor necrosis factor and IL-6 increase and thereby cause an increase of acute phase reactants, including immunosuppressive acidic protein, C-reactive protein and other substances. As a result, postoperative immune suppression is commonly seen in response to surgical stress due to complex interaction of various hormones (particularly adrenal corticosteroids), cytokines, and acute phase reactants.^4^

Laparoscopic surgery should cause less immune impairment, as it is associated with less tissue damage.^5^ On the other hand, during laparoscopy electrosurgical devices are commonly used for hemostasis. The Ligasure ™ (Valleylab Inc., Boulder, CO, USA) vessel sealing instrument and Harmonic scalpel (Ethicon Endo-Surgery, Cincinnati, OH, USA) are the most popular devices for vessel sealing. The Ligasure™ (LS) vessel sealing instruments use a high-current, low-voltage continuous bipolar radiofrequency energy in combination with a feedback controlled response system that automatically delivers and disrupts the power according to the impedance of the tissue between the jaws of the instruments. It fuses collagen and elastin within the vessel walls, resulting in a permanent seal that can withstand three times the normal systolic pressure and seals vessels up to 7 mm.^6^ But Ligasure is not suitable for colpotomy so monopolar current should be used to detach the uterus from the vagina.

The other most popular device used for laparoscopic hysterectomy is Harmonic scalpel and it produces tissue effects by converting electrical energy into vibrations at more than 20,000 cycles per second which is above the audible range. It has approval of United States food and drug administration to seal vessel up to 5 mm in diameter.^6^ But the disadvantage of harmonic scalpel is, it does not reliably seal large vessels, so for large pedicles like infundibulopelvic and uterine artery pedicle, bipolar energy should be used for safety. Safety and efficacy of these newer instruments in laparoscopic surgeries have been studied by many authors comparing different factors like total operative time, blood loss, mean hospital stay, postoperative pain, and postoperative complications but at present, there is lack of evidence regarding the inflammatory stress response with the use of different devices in cases of total laparoscopic hysterectomy.^7^-^9^ This inflammatory stress is very important because energy based surgical devices used for securing vascular pedicles instead of sutures produce collateral thermal damage in neighboring tissue and cause more inflammatory stress.^10^

There are many ways for assessing the postoperative inflammatory response but the measurement of leukocytic changes, including neutrophil- lymphocyte (NLR), platelet-lymphocyte (PLR) ratio, mean platelet volume- lymphocyte ratio (MPVLR) and red cell distribution width- platelet ratios (RPR) are easiest and cheapest methods. White blood cell (WBC), neutrophil, platelet, and lymphocyte counts as well as NLR,PLR,MPVLR and RPR have been well studied in many diseases, such as ulcerative colitis, diabetes, various cancers, surgeries and coronary artery disease.^11^,^12^ But there is not enough information in the literature about how these values change in laparoscopic hysterectomies with *salpingo*-*oophorectomy*. In the current study, we aimed to compare the inflammatory response with alterations in WBC, NLR, PLR, MPVLR and RPR, in patients who underwent laparoscopic hysterectomy and *bilateral salpingo*-*oophorectomy* for benign gynecologic conditions with ligasure plus monopolar cautery or harmonic plus conventional bipolar cautery.

## METHODS

This study was conducted in Tepecik Education and Research Hospital, Obstetrics and Gynecology clinic, which is a reference clinic for Egean region of Turkey. Patients who underwent Laparoscopic hysterectomy with bilateral *salpingo*-*oophorectomy* between January 2017 and January 2022 for benign gynecologic pathology were identified. The records of patients were reviewed retrospectively. Preoperative and postoperative in the first 24 hours hematocrit (HCT), WBC, trombocyt, PLR, NLR, RPR and MPVLR values were compared.

### Inclusion and Exclusion Criteria

Patients with active infection, corticosteroid use, acetylsalicylic acid, and anticoagulant use were not included in the study. Due to animal studies that have shown that oophorectomy changed the leukocyte count by altering the cytokine response we only included patients with bilateral oophorectomy.^13^ We excluded patients whose ovaries were not taken.

Patients were divided into two group, according to instruments used during surgery. Instruments were used according to surgeons preference. Bladder and bowel injuries, blood transfusion requirements, wound infection and hematoma, postoperative respiratory system complications were evaluated as surgical complications and excluded. Maternal venous blood samples were taken into hemogram tubes. The calibrations of the device were completed and analyzed using the Pentra DF Nexus Hematology System® (Horiba Healthcare, Japan). PLR, NLR and MPVLR were calculated by dividing platelet, neutrophil and mean platelet volume counts, respectively, by the lymphocyte count and RPR was calculated by dividing red cell distribution width by the platelet count.

The Student’s t-test and the Mann-Whitney U-test were used to compare the normal and the non-normal distributed quantitative variables, respectively. The paired sample t-test was used for the preoperative and postoperative comparisons of the variables with normal distribution. The Wilcoxon signed-rank test was used for the preoperative and postoperative comparisons of the variables with non-normal distribution. Pearson’s chi-square test and Fisher’s exact test were used to compare the qualitative data. Statistical significance was accepted at p < 0.05.

### Surgical Technique Ligasure with monopolar cautery

All patients were administered general anesthesia and positioned in the low lithotomy position. Total laparoscopic hysterectomy was performed by using one 10 mm umbilical puncture, one 10 mm left upper puncture and two 5 mm punctures for side ports. A RUMI II uterine manipulator with a Koh colpotomy ring and vaginal pneumo occluder balloon was used for manipulating the uterus and delineating the vaginal cuff. The hysterectomy was started with the division of *infundibulopelvic* ligaments. The *infundibulopelvic* ligaments and round *ligament*s were secured and divided by the LigaSure system (Valleylab, Boulder, CO). The vesicouterine peritoneal fold was opened and the bladder was mobilized by blunt and sharp dissection, using Ligasure until the anterior vagina was identified.

The uterine vessels were skeletonized, sealed, and divided by the LigaSure system. The vagina was entered posteriorly first by cutting with a monopolar L-hook at the cervicovaginal junction just above the uterosacral ligament, leaving the supports of the vagina intact.The incision was continued over the colpotomy ring of the uterine manipulator and the uterus was completely detached from the vagina. The uterus was pulled out of the vagina in cases with small uteri, whereas larger uteri were morcellated vaginally. A glove with sponges was placed to maintain the pneumopritoneum, and the vaginal cuff was sutured laparoscopically with a 0-vicryl suture (Ethicon, Somerville, NJ), using a 40-mm round bodied needle. Intermittent mattress suture, taking care to take bites from the angles incorporating uterosacral ligament on both sides, was taken. The Foley catheter was removed after six hours.

### Harmonic with bipolar cautery

*Infundibulopelvic ligament*s and uterine vessels were coagulated using bipolar cautery before divided by harmonic scalpel and colpotomy was done by harmonic scalpel. All other procedures were same with ligasure group. All the cases were done by AGK, ET and AHI.

## RESULTS

**Fig.1:**
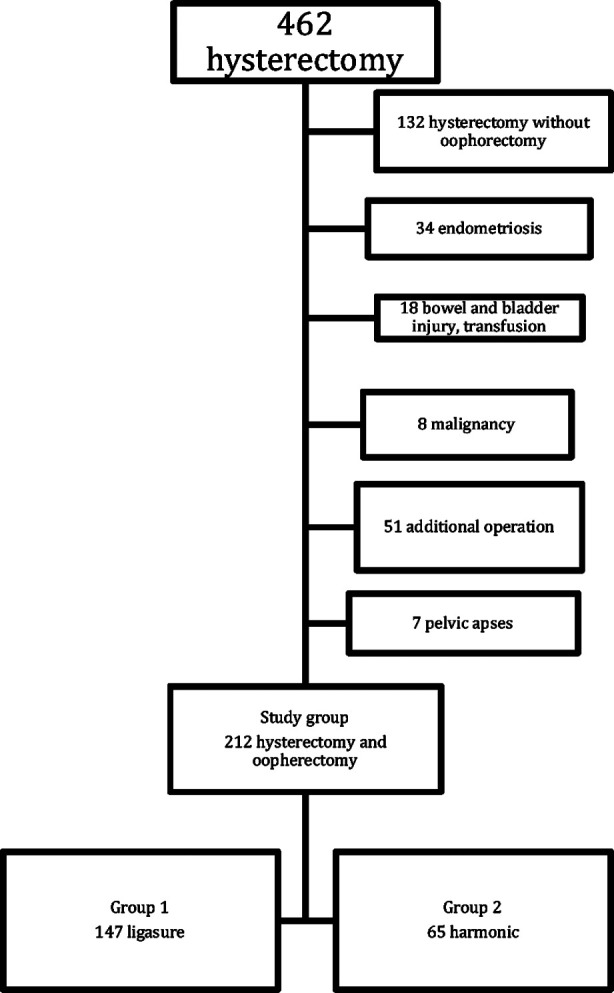
Flowchart of the study.

A total of 462 patients underwent hysterectomy for benign gynecologic pathology. After exclusion, 212 patients were included in the study. The flowchart of the study is seen in Fig.1. In the study group, 147 patients were operated with ligasure plus monopolar electrocauter and 65 with harmonic scalpel plus bipolar electrocautery. The demographic features are shown in Table-I. There were no significant differences between the groups in terms of the demographic data.

**Table-I:** Demographic features of the study group.

	Ligasure plus monopolar (n:147)	Harmonic plus bipolar (n:65)	P value
Age (years)	56.32 ( ± 8.16)	54.65 ( ± 6.97)	0.152
Gravida	2.87 (± 1.67)	3.12 (± 1.83)	0.079
Systemic disease	16 (% 10.8)	9 (%13.8)	0.067
Number of previous perations	62 (% 42.1)	23 (% 35.3)	0.900
***Indication***			
Fibroid	42(% 28.5)	17(% 26.1)	0.326
Brca +	7(% 4.7)	0(% 0)	
hyperplasia	29(% 19.7)	6(% 9.2)	0.153
Ovarian cyst	28(% 19)	25(% 38.4)	0.021
MMR	19(% 12.9)	8(% 12.3)	0.438
PMK	14(% 9.5)	6(% 9.2)	0.409
Cervical dysplasia	8(% 5)	3(% 4.6)	0.387
Duration of hospital stay	1.56 ± 0.6	1.68 ± 0.4	0.253

**Table-II:** Changes in the inflammatory parameters

	Device used	N	Mean	Std. Deviation	P value
Difference in leucocyt count (preop-postop)	ligasure	147	-3027,2109	2043,21657	0.096
	harmonic	65	-2489,2308	2404,82088	
Difference in lymphocyt count (preop-postop)	ligasure	147	89,1156	652,40643	0.001
	harmonic	65	-241,5385	513,22884	
Difference in neutrophly count (preop-postop)	ligasure	147	-2957,1429	2001,64316	0.008
	harmonic	65	-2101,5385	2405,42955	
Difference in hematocrit count (preop-postop)	ligasure	147	4,7259	2,49808	0.726
	harmonic	65	4,5985	2,28278	
Difference in trombocyt count (preop-postop)	ligasure	147	40122,4490	33106,90404	0.546
	harmonic	65	37200,0000	30902,66979	
Difference in plr count (preop-postop)	ligasure	147	10,551497	56,7784902	0.001
	harmonic	65	35,915538	38,3638379	
Difference in nlr count (preop-postop)	ligasure	147	-2,159252 -	2,4535339	0.001
	harmonic	65	,805846	1,4503231	
Difference in rpr count (preop-postop)	ligasure	147	-,011327 -	,0088024 ,	0.094
	harmonic	65	,009185	0079587	
Difference in mpvlr count (preop-postop)	ligasure	147	-,459592	1,9090268 ,	0.001
	harmonic	65	,530615	9056384	

The most common indication in the ligasure group was fibroid, whereas the most common indication in the harmonic group was ovarian cyst. Regardless of the procedure, WBC and RPR count increase, hematocrit and trombocyt decrease in both group but the difference is not statistically significant. Lymphocyt count decrease in ligasure group and increase in harmonic group. (P:0,001). Neutrophyl and NLR count increase in both group, but in the ligasure group the increase is statistical significant then harmonic group (P:0,008 and P:0,001). PLR count decrease in both group, but in the harmonic group the decrease is statistical significant then ligasure (P:0,001). MPVLR count increase in ligasure group and decrease in harmonic group (P:0,001).

In the postoperative period, the inflammatory markers lymphocyte count, neutrophyl, NLR, PLR and MPVLR count changed less in the harmonic plus bipolar cautery group which shows less inflamatuar response in this group.

## DISCUSSION

Cell damage due to surgical trauma causes the release of endogenous mediators. These mediators are called damage associated molecular patterns or alarmins.^14^ Alarmins are recognized by Toll like receptors expressed by dendritic cells and macrophages. Activation of Toll like receptor signaling pathway causes mobilization of large quantities of immature neutrophils from the bone marrow into circulation which causes neutrophily and leucocytosis.^15^ Surgical trauma is associated with an increase in the leukocytes count, but also with a decrease in the number of CD4+ and CD8+ lymphocytes. One of the mechanisms responsible for the decrease in the number of lymphocytes are disturbances in the mechanisms regulating apoptosis of these cells. Beyond these, there are a lot of changes in *complete blood count parameters* and derivatives after surgery. Due to the combined effects of hemodilution and accelerated platelet consumption, platelet count is decreased. The more decrease in platelet count shows more surgical trauma.

The platelet-lymphocyte ratio (PLR) is a novel inflammatory marker and recent studies show that a high PLR reflects inflammation.^16^ Neutrophil-lymphocyte ratio (NLR) is used as a marker of inflammation. A higher ratio, indicative of a hyperinflammatory situation.^17^ Red cell distribution width (RDW), an indicator of size variability among circulating red blood cells, has gained considerable attention as an inflammatory marker.^18^,^19^ A new index, the ratio of RDW to platelet count (RPR), has been reported to reflect the severity of inflammation.^20^ A higher ratio, indicative of an hyperinflammatory situation. Mean platelet volume (MPV) is an indicator of platelet activation and aggregation.MPV-lymphocyte ratio (MPVLR) have been shown to be important indicator of systemic inflammation.^21^ Higher MPVLR indicative of an hyperinflammatory situation.

Since laparoscopic surgery is associated with less surgical trauma than conventional surgery, it can be assumed that it also causes less trauma related inflammation .But electrosurgical devices used in laparoscopy inevitably cause varying degrees of thermal spread and more inflammation than knot tying surgery. There are a lot of electrosurgical devices used in laparoscopy. Studies have evaluated the different devices in terms of their safety, efficacy, extent of thermal injury, and versatility; however, none of the devices has been proven superior over another.^22^,^23^ In the laparoscopic hysterectomy surgical technique, the vaginal cuff should be cut with a monopolar cautery or harmonic scalpel. Because of this, generally during laparoscopic hysterectomy, Ligasure plus monopolar cautery or harmonic plus bipolar cautery combination is used. Since they are the most frequently used devices, we compare this two surgical device group in terms of inflammatory markers which is inexpensive and easy to measure.

We found that, base on the laboratory findings, the inflammatory markers change more in Ligasure plus monopolar cautery group compared with harmonic plus bipolar cautery group. Similarly, Kallol et al. showed that Ligasure produced a more sustained and greater inflammatory response at 24 hours postsurgery.^5^ Our results also showed that Ligasure plus monopolar cautery group cause more inflammatory response but it is not possible to determine which of the Ligasure or monopolar cautery caused this difference.

### Limitation

The main limitation of our study is its retrospective nature but the reliability of our medical records minimizes this limitation. The main strength of our study is large sample size and also the homogeneity between two groups, makes our result more reliable. There are many studies in literature that compare different energy devices but to our knowledge, this is the first study that compare the impact of combination of electrosurgical devices (Ligasure plus monopolar cautery or harmonic plus bipolar cautery combination) on the inflammatory markers.

## CONCLUSION

Ligasure plus monopolar cautery group compared with harmonic plus bipolar cautery group cause more inflammatory changes in complete blood count. However, further studies are needed to show whether these changes in laboratory findings affect clinical situations.

### Authors Contribution:

**ET:** Project development, data acquisition, data analysis and manuscript writing, is responsible for integrity of research.

**AGK** and **AHI:** Did project development, data acquisition, data analysis and manuscript editing.

**IK** and **SYK**: Project development, data acquisition, data analysis and manuscript writing.

**AD:** Did manuscript editing, data collection and data analysis.
